# Liver Transplantation for Hepatocellular Carcinoma: A Single Center Resume Overlooking Four Decades of Experience

**DOI:** 10.1155/2016/7895956

**Published:** 2016-01-10

**Authors:** Nikos Emmanouilidis, Rickmer Peters, Bastian P. Ringe, Zeynep Güner, Wolf Ramackers, Hüseyin Bektas, Frank Lehner, Michael Manns, Jürgen Klempnauer, Harald Schrem

**Affiliations:** ^1^Department of General, Visceral and Transplant Surgery, Hannover Medical School, Carl Neuberg Strasse 1, 30625 Hannover, Germany; ^2^Department of Gastroenterology, Hepatology and Endocrinology, Hannover Medical School, Carl Neuberg Strasse 1, 30625 Hannover, Germany; ^3^IFB-TX Core Facility and HTA, Hannover Medical School, Carl Neuberg Strasse 1, 30625 Hannover, Germany

## Abstract

*Background.* This is a single center oncological resume overlooking four decades of experience with liver transplantation (LT) for hepatocellular carcinoma (HCC).* Methods.* All 319 LT for HCC that were performed between 1975 and 2011 were included. Predictors for HCC recurrence (HCCR) and survival were identified by Cox regression, Kaplan-Meier analysis, Log Rank, and *χ* ^2^-tests where appropriate.* Results.* HCCR was the single strongest hazard for survival (exp⁡(*B*) = 10.156). Hazards for HCCR were tumor staging beyond the histologic MILAN (exp⁡(*B*) = 3.645), bilateral tumor spreading (exp⁡(*B*) = 14.505), tumor grading beyond G2 (exp⁡(*B*) = 8.668), and vascular infiltration of small or large vessels (exp⁡(*B*) = 11.612, exp⁡(*B*) = 18.324, resp.). Grading beyond G2 (exp⁡(*B*) = 10.498) as well as small and large vascular infiltrations (exp⁡(*B*) = 13.337, exp⁡(*B*) = 16.737, resp.) was associated with higher hazard ratios for long-term survival as compared to liver transplantation beyond histological MILAN (exp⁡(*B*) = 4.533). Tumor dedifferentiation significantly correlated with vascular infiltration (*χ* ^2^ *p* = 0.006) and intrahepatic tumor spreading (*χ* ^2^ *p* = 0.016).* Conclusion.* LT enables survival from HCC. HCC dedifferentiation is associated with vascular infiltration and intrahepatic tumor spreading and is a strong hazard for HCCR and survival. Pretransplant tumor staging should include grading by biopsy, because grading is a reliable and easily accessible predictor of HCCR and survival. Detection of dedifferentiation should speed up the allocation process.

## 1. Introduction

The repertoire of treatment strategies for hepatocellular carcinoma (HCC) consists of liver resection (LR), chemotherapy (CTX), radio frequency ablation (RFA), transarterial chemoperfusion (TACP), selective internal radiation therapy (SIRT), transarterial chemoembolisation (TACE), percutaneous ethanol instillation (PEI), monoclonal antibody therapy (mAB), and liver transplantation (LT).

**Figure 1:**
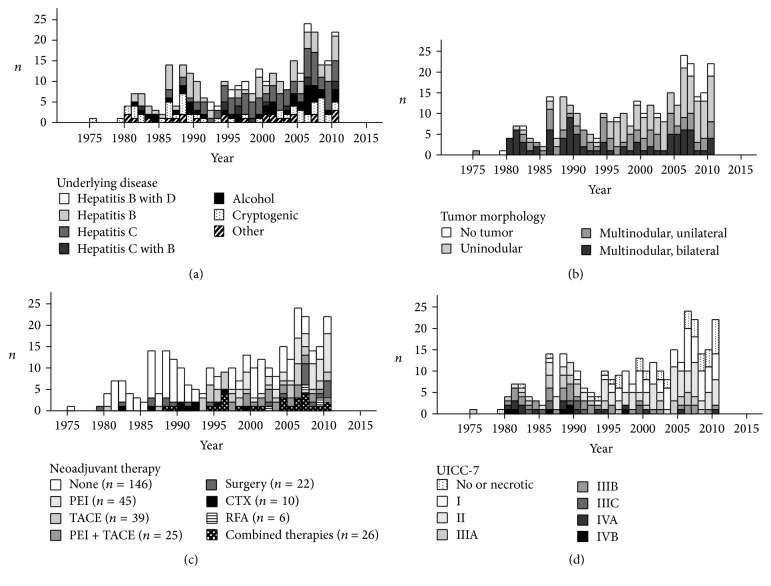
Annual proportions of underlying diseases (a), neoadjuvant therapies (b), UICC-7 staging (c), and tumor morphologies (d). (a) There was no significant change in annual proportions of recipients underlying diseases over time. (b) Tumor morphologies of transplanted HCC changed over time in the favour of uninodular and unilateral tumors. (c) The overall rate for neoadjuvant therapy as well as the diversity of different treatment combinations increased over time. This effect was caused due to new therapies that were introduced consecutively from 1975 to 2010* (surgery (S), chemotherapy (CTX), transarterial chemoembolisation (TACE), percutaneous ethanol instillation (PEI), selective internal radiation therapy (SIRT), and monoclonal antibodies (mAB))*. (d) Between 1975 and 2010 the proportion of low graded UICC-7 staged tumors increased significantly.

The first elective liver resections were performed in the late 19th century [1–3], but although Wendel [4] already performed a successful anatomic right hemihepatectomy for a HCC in 1911, it took another 50 years and a better understanding of the liver anatomy [5] before liver resections were performed on a larger scale by multiple centers worldwide [6–10]. The first liver transplantation for a “hepatoma” was the second LT that was published in the pioneering report by Starzl et al. in 1963 [11]. A decade later Cyclosporin [12] was introduced as a new immunosuppressant and in the following years larger series of liver transplantations were accumulated [13, 14]. The early survival analyses of LT for HCC though were rather disappointing [15] with 2-year survival rates of 25–30% compared to 70% for benign diseases [16, 17]. Those disappointing results ignited the development of nonsurgical treatment alternatives for HCC: starting with systemic chemotherapy and transarterial chemoperfusion [18] on an experimental scale in the early 1980s. A decade later SIRT [19], TACE [20, 21], and PEI [22] were introduced and another ten years later RFA [23] was added (Figure 1(c)). The latest development was the introduction of monoclonal antibody therapy in 2008 [24, 25].

Covariates which possibly affect HCC recurrence (HCCR) and survival after LT are underlying liver disease [26], tumor size [27], grading [28], tumor multifocality, vascular invasion [26, 29], *α*-fetoprotein [30], and adjuvant or neoadjuvant therapy [27, 31, 32]. But despite extensive and long experience with LT for HCC there are very few reports with follow-up data of more than a decade [13, 33–36]. Most long-term reports cover only 5 years of follow-up [27, 28, 32, 37–43].

Here we report our long-term single center experience of more than four decades with all consecutive patients (*n* = 319) who received LT for HCC between 19th November 1975 and 12th December 2010. The main focus of this study was the oncological long-term aspects and the value of liver transplantation for the treatment of HCC.

## 2. Patients and Methods

### 2.1. Patients

Diagnosis of HCC was verified before LT and/or at the histological examination of the explanted liver (*n* = 319). The mean follow-up was 6.4 years (median 4.8 years, range 0.2 to 30.9 years). Follow-up with respect to time from last contact to query in relation to time of LT to query was completed in 96% (median 100%, range 4 to 100%). Time span of last contact to query in living patients was 0.5 to 29.4 years (median 5.9 years).  Table 2 summarizes the clinical data of the investigated cohort.

### 2.2. Immunosuppressive Therapy

Early transplantations were performed under protection with Azathioprine and Corticosteroids medication. Next step in immunosuppressive evolution was the introduction of the Calcineurin-inhibitor Cyclosporin A (CsA). Combinations of CsA with Corticosteroids and even triple therapies with CsA, Azathioprine, and Corticosteroids were applied. Then FK-506—another Calcineurin-inhibitor—was introduced and added to the portfolio of immunosuppressants. The combination of FK-506 with Corticosteroids was a common replacement therapy for the standard protocol of CsA plus Corticosteroids. Azathioprine was only scarcely used, until it completely disappeared as a standard medication in solid organ transplantation. Another significant improvement was the introduction of Mycophenolate Mofetil, which was mainly used as a triple supplement in order to reduce the dosage of Calcineurin-inhibitor medications, because it was realized that the Calcineurin-inhibitor nephrotoxicity was a significant problem in the long run. Other additional immunosuppressants in recent years were the mTOR inhibitors sirolimus (Rapamycin) and everolimus (RAD-001) and the CTLA-4 antibody belatacept (LEA29Y). The latter ones were applied mainly as study drugs within multicenter trials and thus were not commonly used. Overall, the high level of diversity in applied immunosuppressive therapies in this cohort of patients not only is caused by the number of different immunosuppressants and their combinations but is even more diversified due to different dosages and even therapy changes in individual patients during follow-up.

Today's standard treatments in liver transplantation at our facility consist of Corticosteroids (prednisolone, methyl-prednisolone), basiliximab (only perioperatively), Mycophenolate Mofetil, and the Calcineurin-inhibitor FK-506.

### 2.3. Tumor Morphology, UICC-7 Staging, and “Inside/Outside” hMILAN Categorization

All tumors were retrospectively restaged according to the pathohistological examination of the explanted liver and following the 7th edition of the UICC classification (*UICC-7*). For tumor morphology we also categorized each tumor into either nondetectable, uninodular, multinodular/unilateral or multinodular/bilateral intrahepatic tumor spreading. This categorization as well as the categorization referring to MILAN criteria was done on the basis of the histopathological reports in order to circumvent the otherwise unavoidable bias by the technological development of imaging techniques during the last forty years. The retrospective classification either as “inside” or as “outside” MILAN was defined as* histological MILAN* (*hMILAN*). The preoperative MILAN classification, which is usually commonly applied for the listing of HCC patients and carried out by imaging technologies, is renamed* iMILAN* for discrimination purposes.

### 2.4. Survival Data und HCC Recurrence (HCCR)

HCCR and survival were checked in close cooperation with the German national cancer registry and the German national address registry and by continued follow-up in our outpatient transplant clinics. Data were complemented by targeted interviews of referring physicians if necessary. Descriptive statistics related to HCC recurrence and HCC recurrence related deaths are summarized in Tables 3 and 4.

### 2.5. Statistical Analysis

Statistical analyses were performed using SPSS v23 (PASW Statistics Inc., IBM, Somers, NY, USA). *p* values and hazards for survival and HCC recurrence (HCCR) were calculated by multi- or univariate Cox regression. Covariate hazards of survival were* underlying disease, UICC-7 staging, hMILAN status, vascular infiltration, neoadjuvant therapy,* and* grading. HCCR* as a hazard for survival was included as a time-dependent covariate. Covariate hazards for HCCR were* underlying disease*,* UICC-7 staging*,* hMILAN status*,* vascular infiltration*,* neoadjuvant therapy, *and* grading. p* values below 0.05 were defined as significant. Hazards (exp⁡(*B*)) > 1.0 indicated a higher risk and hazards (exp⁡(*B*)) < 1.0 indicated lower risk for HCCR or death. Survival data and HCCR data were graphically plotted using Kaplan-Meier statistics. Comparison of cohort identifiers was performed using a *χ* ^2^-test.

## 3. Results

### 3.1. Descriptive Statistics

Table 2 shows the descriptive statistics of the population of all *N* = 319 patients that had been transplanted with the diagnosis of HCC between 1975 and 2010. Mean age at time of LT was 51.0 years (±SD 12.5) with a median of 54.1 and a male-to-female ratio of 3 : 1. Predominant underlying diseases were hepatitis C (*n* = 86; 27%), hepatitis B (*n* = 85; 27%), hepatitis B with D (*n* = 15; 5%), hepatitis C with B (*n* = 12; 4%), alcohol (*n* = 47; 15%), and cryptogenic cirrhosis (*n* = 50, 16%). Neither NAFLD (nonalcoholic fatty liver disease) nor NASH (nonalcoholic steatohepatitis) was a standard terminology used for enlisting patients for LT at our transplant center. But it can be assumed that the group of cryptogenic cirrhosis also includes those forms of cirrhosis. Other underlying diseases or codiseases (*n* = 24; 8%) were juvenile hepatoblastoma, adenomatosis, hypertyrosinemia, Wilson's disease, hemochromatosis, *α*1 antitrypsin deficiency, Budd Chiari syndrome, androgen therapy, biliary cirrhosis, autoimmune hepatitis, and chronic lead intoxication (Table 2). There was no significant change in the category of underlying diseases over time (Figure 1(a)). Most HCC tumors had a multinodular morphology (*n* = 166; 52%). This category of multinodular tumors was divided into multinodular/unilateral tumors (*n* = 79; 25%) and multinodular/bilateral tumors (*n* = 87; 27%). Uninodular HCCs were observed in *n* = 133 (42%) patients. There was also a significant proportion of pretreated patients in whom no HCC could be detected at the histological examination of the explanted recipients livers (*n* = 20; 6%). The largest tumor had a volume of 14137 cm^3^ and the smallest tumor had a volume of 2 cm^3^ (mean = 320 cm^3^, median = 31.4 cm^3^). AFP measured before LT had a range from 0 to 214975 ng/mL (mean = 2513 ng/mL, median = 21 ng/mL). Living related transplantations were performed in *n* = 12 (4%) recipients. Split-liver transplantations were performed in *n* = 19 (6%) patients and partial/reduced size transplantations in *n* = 13 (4%) patients. Cold ischemic time ranged from 100 to 1970 minutes (mean = 624 minutes, median = 611 minutes). Twenty-nine patients (9.1%) received a second LT and one patient received an additional third LT. Two patients were retransplanted after diagnosis of intrahepatic HCCR, which occurred at 5.7 and 8.8 years after primary liver transplantation. Time from HCCR to retransplantations was 61 and 499 days, respectively. One patient is still alive with a tumor-free survival after second LT of 18.7 years. The second patient died at 2.2 years after second LT due to multilocal 2nd HCCRs at lungs, liver, and abdominal wall and with a peritoneal seeding.

All other retransplants were not related to HCCR. From 1975 to 2010 by and by several HCC pretreatments were developed* (surgery (S), chemotherapy (CTX), transarterial chemoembolisation (TACE), percutaneous ethanol instillation (PEI), selective internal radiation therapy (SIRT), and monoclonal antibodies (mAB))* and the overall rate of patients who were pretreated before LT and the diversity of treatment combinations increased synchronously (Figure 1(c)). The number of advanced multinodular HCCs and tumors with intrahepatic bilateral spread declined significantly over the years in favour of singular node HCCs (Figure 1(b)) and the proportion of successfully pretreated HCC (tumor necrotic, no tumor detectable) increased (Figure 1(d)).

**Figure 2:**
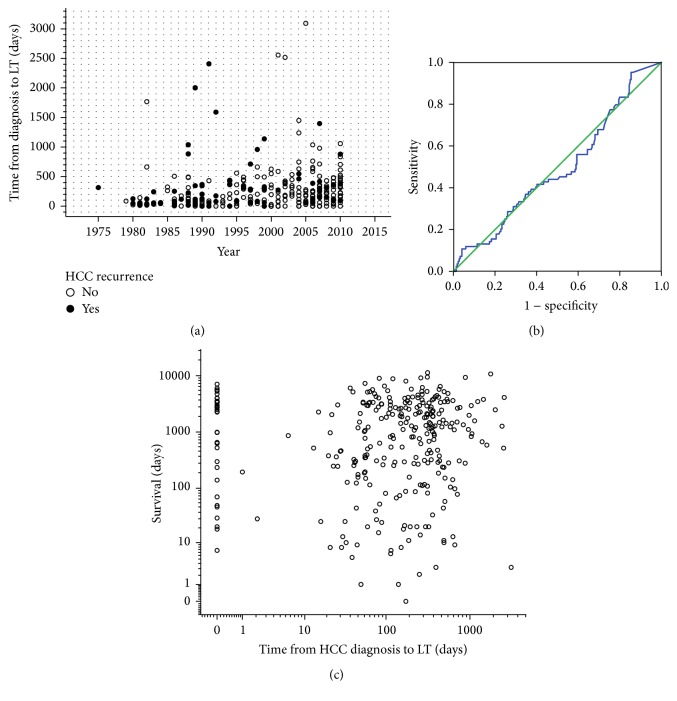
Development of waiting time (b) from 1975 to 2010 and prognostic impact of waiting time on HCC recurrence (b) and overall survival (c). Waiting time increased slightly from about 2-3 months in the early 1980s to an average of 411 days in 2010, but this increase had no significant prognostic impact on HCC recurrence (ROC AUC = 0.494; *χ* ^2^ *p* = 0.319) and overall survival (*σ* ^2^ *p* = 0.279).

*Waiting time* (time from HCC diagnosis to LT) increased slightly during the decades, but this had no significant influence on HCC recurrence or survival (ROC AUC = 0.494; *χ* ^2^ *p* = 0.319; *χ* ^2^ *p* = 0.279, resp.) (Figures 2(a)–2(c)).

In 285 patients HCC diagnosis was known prior to LT, while in 34 patients the diagnosis of HCC was coincidental. 173 patients were pretreated before LT by surgery (*n* = 22), TACE (*n* = 39), RFA (*n* = 6), PEI (*n* = 45), CTX (*n* = 10), or combinations of each (*n* = 41) (Figure 4).* PEI*,* TACE*, and surgery represented the dominant choices of pretreatment strategies. The tumor response to mono- or multimodal neoadjuvant therapies is shown in Figure 4(b).* PEI* and* TACE* were comparable in terms of remaining vital tumor tissue (Fisher's exact test *p* = 0.439). Therapy efficacy though was not comparable one-on-one because of a significant higher overall proportion of multinodular tumors in the* TACE* group and different proportions of multinodular/bilateral HCCs, which was three times as high for the* TACE* groups as compared to the* PEI* group (26% to 9%) (Figure 4(a)). Neoadjuvant therapy by* surgery* resulted in the highest rate of nondetectable tumors (45%) (10 of 19) (Figure 4(b)), but this difference was statistically not significant compared to the proportion of full-necrotic plus nondetectable tumors of the* PEI* group (Fisher's exact test *p* = 0.099). 178 patients (56%) were transplanted* inside* and 141 (44%) were transplanted* outside hMILAN*. Prior to the introduction of MILAN criteria (1997) 82 patients (65%) had been transplanted* outside* and 43 patients (34%)* inside hMILAN*. After 1997 59 patients (30%) were transplanted* outside* and 135 (70%)* inside hMILAN*. 16 (38%) of the 42 survivors who lived longer than 10 years and 6 of the 9 recipients (67%) who lived longer than 20 years after LT were transplanted* outside hMILAN*. Only one of those patients died, but not due to HCCR.

In order to have a clear analysis on HCC recurrence relevant data we censored all patients with perioperative hospital mortality (*n* = 68; 21%) (Table 1), who as a matter of course did not survive long enough for developing any HCCR. Eighty-three (*n* = 83; 33%) of the remaining 251 patients were diagnosed with HCCR during follow-up. Most HCCRs were solely extrahepatic tumor recurrences (*n* = 48; 58%). In 15 patients (18%) HCCR was diagnosed as exclusive* intrahepatic* tumor recurrences. In 20 patients (24%) HCCR was synchronously found in intra- and extrahepatic locations. In 34% (*n* = 30) of HCC recurrences metastases were found in more than one anatomic location. Dominant site of* extrahepatic* HCCR was the lung (*n* = 34), followed by bone (*n* = 13), lymph nodes (*n* = 9), and brain (*n* = 7) (Table 3). Sixty-three (*n* = 63; 76%) of the patients with HCC recurrences died due to this tumor recurrence and *n* = 52 (21%) patients died due to non-HCCR related reasons (Tables 1 and 4). Cox regression analysis was performed in order to calculate the odds ratios (exp⁡(*B*)) and significance levels of the tested covariates for their risk to be associated with HCC recurrence (Table 5). For a clear view on the prognostic oncological value of LT we had to purge the cohort of patients further by censoring any causes of death other than HCC recurrence related ones and analysed the cumulative survival rates of the remaining *n* = 199 patients with respect to the selected covariates (Tables 1 and 6; Figures 5(b), 7(a)–7(d), and 9(a)–9(f)). Thus, *n* = 9 patients with diagnosis of HCCR, but with mortality due to other reasons, were excluded from this analysis.

### 3.2. Survival and HCC Recurrence

**Figure 3:**
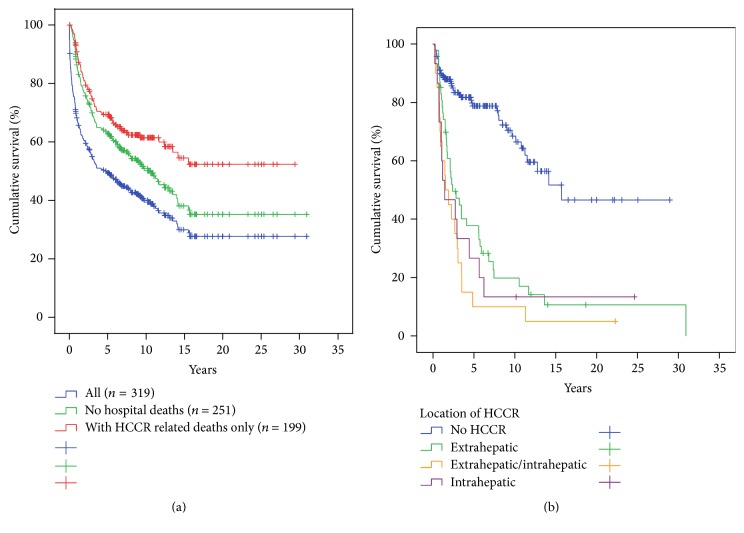
Survival with respect to hospital mortality and HCC recurrence. (a) Cumulative survival of all patients (*n* = 319,* blue line*), without hospital mortality (*n* = 251,* green line*) and with HCC recurrence related deaths only (*n* = 199,* red line*). (b) HCC recurrence-free survival (blue line, Cox regression analysis with HCC recurrence as time-dependent covariate) and with respect to extrahepatic (green line), intrahepatic (red line), or combined extrahepatic/intrahepatic HCC recurrences (orange line). HCC recurrence was highly significant hazard of survival (*p* < 0.001, exp⁡(*B*) = 10.156), but it made no difference to survival whether HCC recurrences were at intrahepatic, at extrahepatic, or at combined intrahepatic/extrahepatic locations (*σ* ^2^ *p* > 0.05).

**Figure 4:**
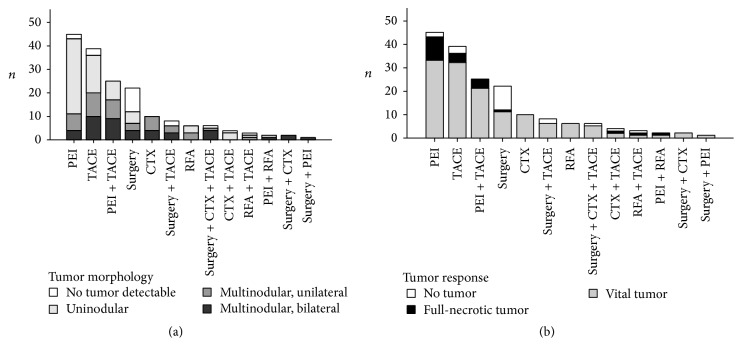
HCC morphology per treatment group (a) and tumor response to pretreatments (b) as measured in numbers of nondetectable, full-necrotic, or vital tumors. Percutaneous ethanol instillation (PEI) (*n* = 45), transarterial chemoembolisation (TACE) (*n* = 39), and surgery (*n* = 22) were most frequently applied. Another major treatment group were patients that had been treated by a combination of* PEI* and* TACE* (*n* = 25). There were a significant higher number of uninodular tumors in the* PEI* group (71%) as compared to the* TACE* group (41%). The* TACE* group also had a significant higher proportion of multinodular tumors (52%) as compared to the* PEI* group (25%) and a higher proportion of multinodular/bilateral tumors, which was three times as high as compared to the* PEI* groups (26% to 9%, resp.). The pretreatment group* surgery* had the highest rate (45%) (10 of 22) of explanted livers without detectable tumor remnants, but this difference was statistically not significant as compared to the proportion of full-necrotic and nondetectable tumors (*n* = 10 + 2) in the PEI group (Fisher's exact test *p* = 0.099). The PEI group and TACE group were comparable in terms of remaining vital tumor tissue (Fisher's exact test *p* = 0.439).

Figure 3(a) shows the Kaplan-Meier plots for the cumulative survival of all patients (*n* = 319) (*blue line*), with hospital mortality excluded (*n* = 251) (*green line*) and with HCC recurrence related deaths only (*n* = 199) (*red line*).

**Figure 5:**
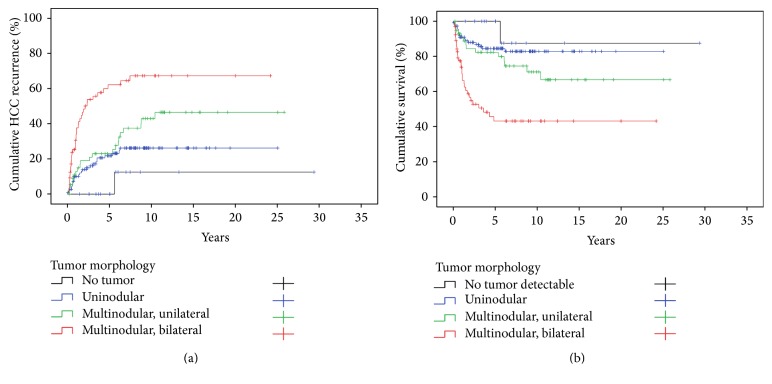
HCC recurrence (a) and survival (b) with respect to tumor morphology. HCC recurrence (hospital deaths excluded) (a) was significantly influenced by tumor morphology (Log Rank *p* < 0.001). Survival (hospital deaths and non-HCC recurrence related deaths excluded) (b) was significantly influenced by the intrahepatic tumor dissemination of the primary HCC (Log Rank *p* < 0.001).

**Figure 6:**
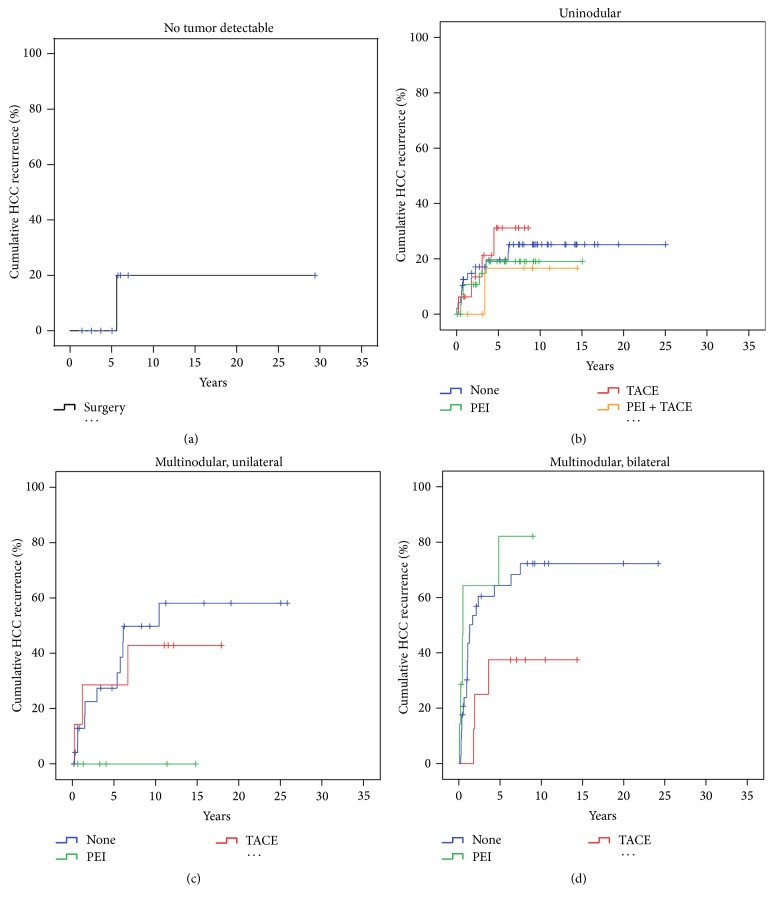
HCC recurrence with respect to tumor morphology and neoadjuvant therapy. The effectiveness as estimated by rate of HCC recurrences was analysed with respect to different neoadjuvant therapy regimen and tumor morphology. Hospital deaths and treatment groups with *n* < 5 were excluded from analysis. Thus, only* surgery* remained for estimation of cumulative HCC recurrence in the group of* nondetectable tumors* (a). In the group of* uninodular* HCC (b) there was no significant difference in HCC recurrence rates comparing the mono- and multimodular pretreatments.* Multinodular/unilateral* HCC (c) had a significantly lower rate of HCC recurrence (Log Rank *p* < 0.001) if treated by* PEI*, while* TACE* did not make a difference for this group of tumors at all (Log Rank *p* > 0.05). In* multinodular/bilateral* tumors (d)* TACE* was significantly better as compared to* PEI* (Log Rank *p* < 0.05). The PEI group had the same cumulative rate of HCC recurrence as the no-treatment group.

**Figure 7:**
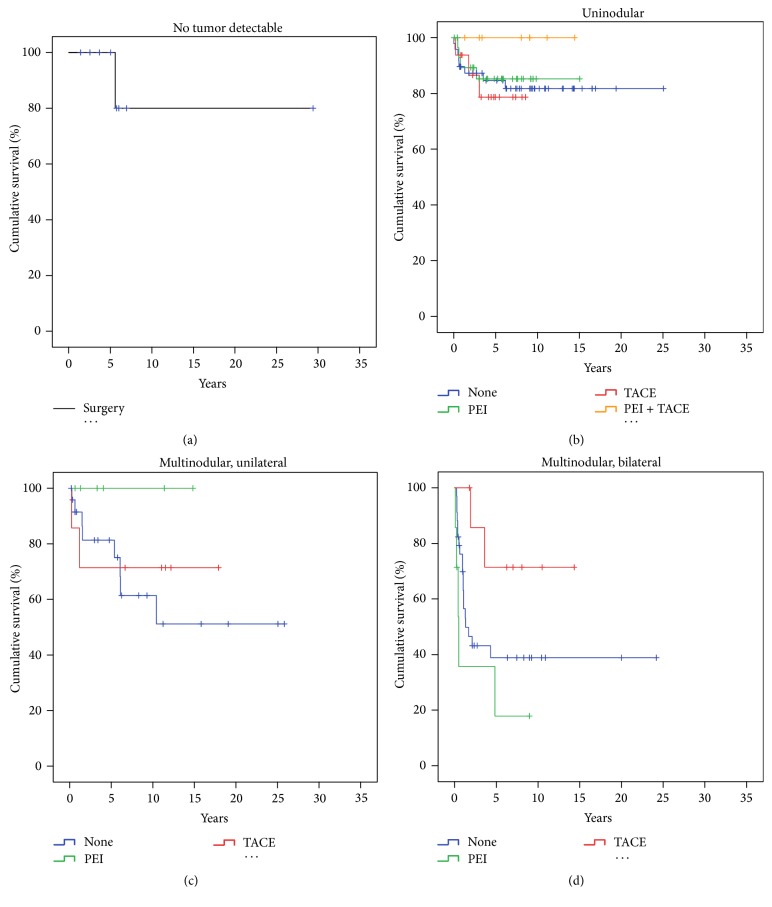
Survival with respect to tumor morphology and neoadjuvant therapy. Hospital mortality and non-HCC recurrence related deaths as well as treatment groups with *n* < 5 were excluded. In the category of* nondetectable tumors* only* surgery* remained with *n* > 5. The cumulative survival in this subcategory was 80% (a). In the category of uninodular HCC (b) there was no difference in survival comparing patients that had been pretreated by* PEI* or* TACE*. For the combination of* PEI* and* TACE* a significantly better survival was observed (Log Rank *p* < 0.05) as compared to* PEI* or* TACE* alone. For* multinodular/unilateral* HCC (c)* TACE* did not make a difference, while pretreatment with* PEI* achieved a significant better survival (Log Rank *p* < 0.05). In* multinodular/bilateral* tumors (d) survival was significantly better for the group of patients who were pretreated with TACE as compared to PEI or no pretreatment.

**Figure 8:**
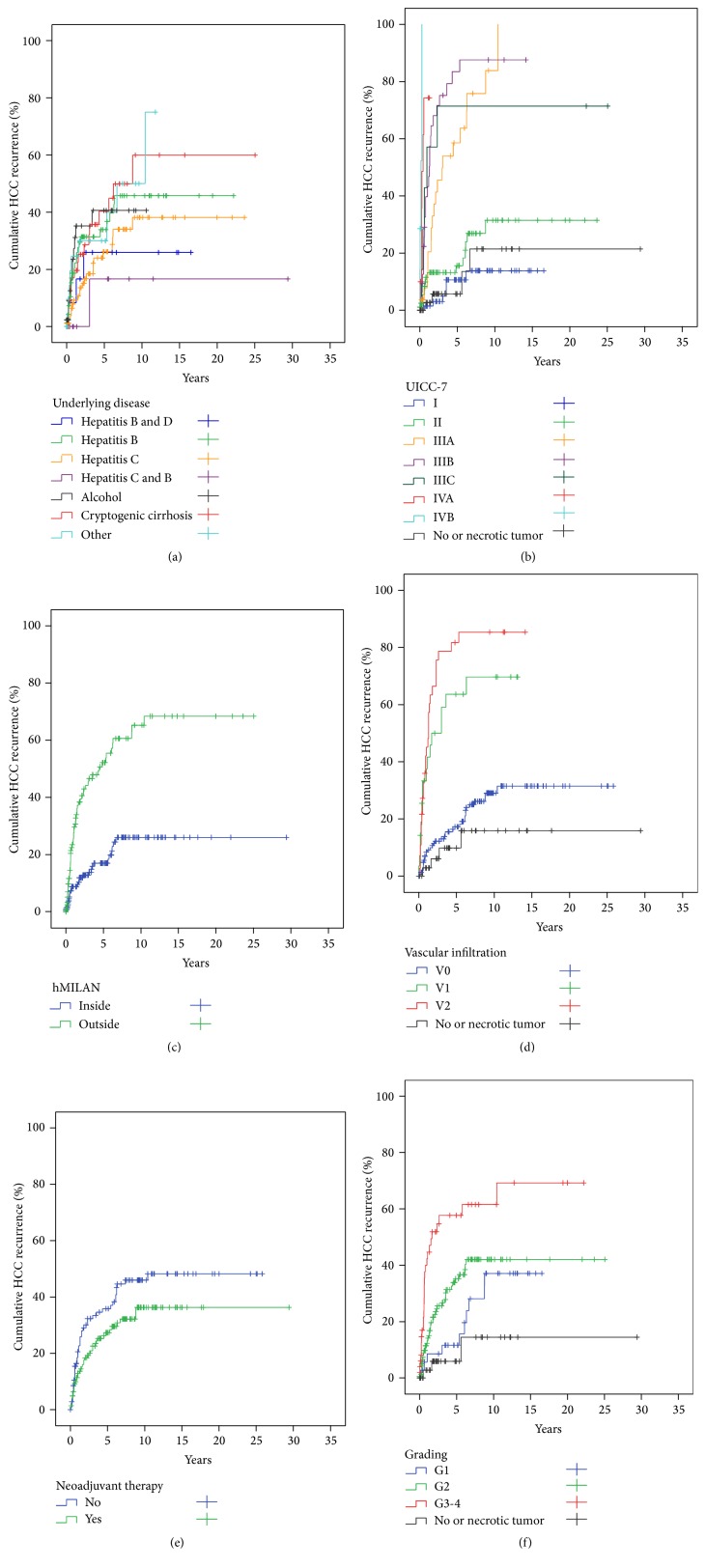
Cumulative recurrence of HCC (hospital mortality excluded) (*n* = 251) (for statistics see Table 3). (a)* Underlying disease* had no significant impact on HCC recurrence. (b)* UICC-7* staging had a significant impact on HCCR. Only UICC I and II staged tumors were comparable to the reference category of* no or necrotic* tumors, while tumors of UICC-7 IIIA-IVB had significantly higher rates of HCCR. (c) The group of patients transplanted outside the histologic MILAN (*hMILAN*) had a maximum cumulative HCC recurrence rate of almost 70% at 10.4 years after LT, while patients transplanted inside* hMILAN* (*reference category*) only had a maximum cumulative HCC recurrence rate of about 25% at 7 years after LT. (d)* Vascular infiltration* was a highly significant predictor of HCC recurrence, while tumors without vascular infiltration had a comparable HCC recurrence rate compared to the reference group of* no or necrotic* tumors. (e)* Neoadjuvant therapy* had no significant impact on HCC recurrence. (f)* Tumor grading* was a significant hazard for HCC recurrence. G1 staged tumors had a comparable risk for HCC recurrence to the reference category (*no or necrotic tumors*), while G2 and G3-4 staged tumors were strong significant hazards for HCC recurrence.

**Figure 9:**
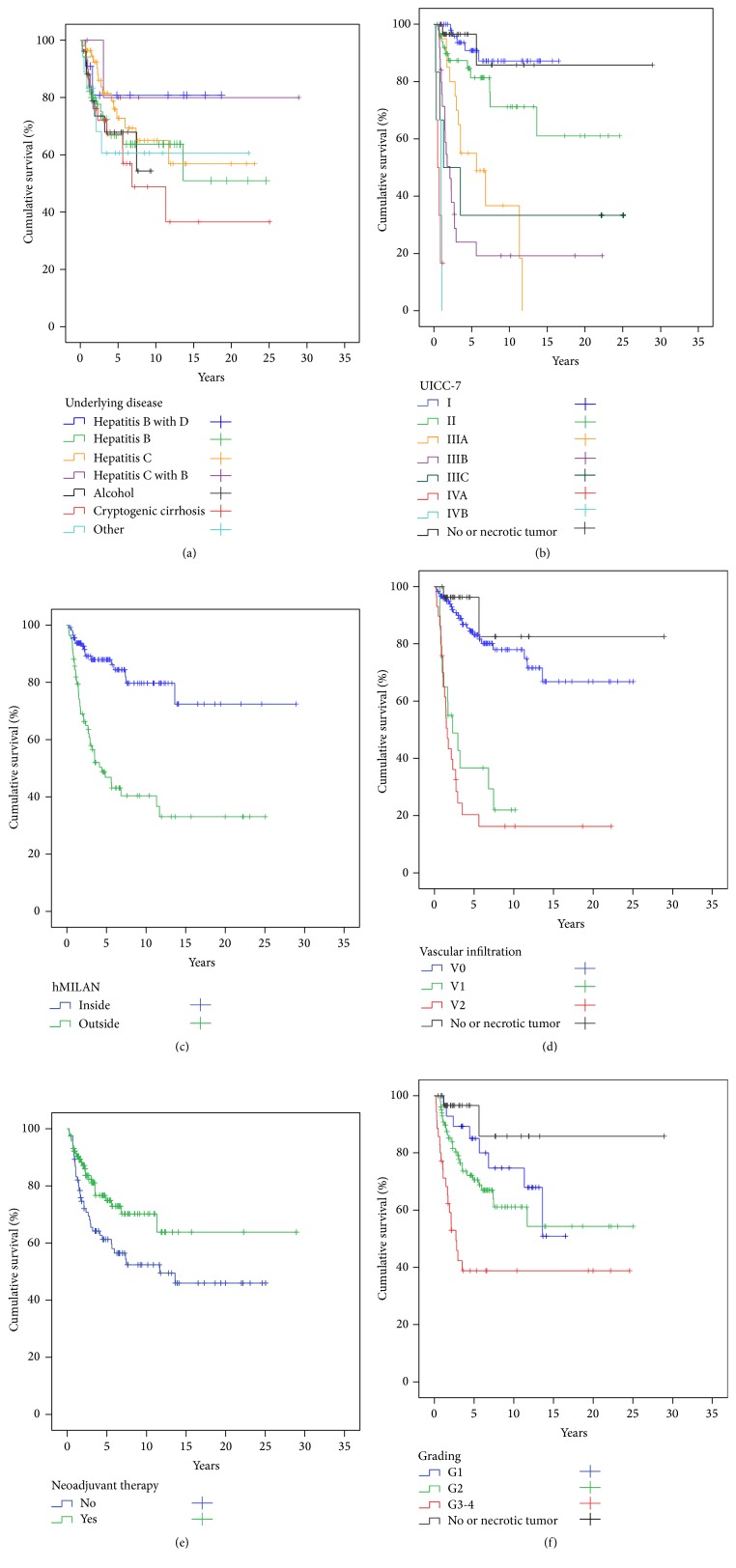
Cumulative survival after LT for HCC (HCC recurrence related deaths only) (*n* = 199) (for statistics see Table 4). (a) With the exception of a better survival comparing the* hepatitis C* versus* cryptogenic cirrhosis* subcategories there were no other significant differences for survival related to* underlying diseases*. (b) Survival for UICC I and II staged tumors was comparable to the reference category (*no or necrotic tumors*), while the risk for HCC recurrence death increased significantly and equivalently with each step of UICC-7 staging above IIIA. (c) Tumors outside the histologic MILAN were significant hazards for survival. Nevertheless, even in the group of patients transplanted outside the histologic MILAN (*hMILAN*) the cumulative survival was 30% at 25 years after liver transplantation. The cumulative survival of patients who were transplanted inside the histologic MILAN (*hMILAN*) was 72% at 30 years after liver transplantation. (d) Small (V1) and large (V2)* vascular infiltration* were significant hazards for a HCC recurrence related death, while tumors without (V0)* vascular infiltration* were no significant hazards for survival compared to the reference category of* no or necrotic tumors*. (e)* Neoadjuvant therapy* in general decreased the HCC recurrence related death rate significantly. (f)* Tumor grading* was a significant predictor of survival. While G1 staged tumors had no increased risk for HCC recurrence related death compared to the reference category (*no or necrotic tumors*), G2 and G3-4 graded tumors were identified as significant hazards for HCC recurrence related deaths. The risk to die from HCC recurrence after liver transplantation was twice as high for G3-4 tumors as compared to G2 graded tumors.

The maximum cumulative rate for HCCR was 33% (83/251) and was reached at 10.4 years after LT. There were no time-dependent differences for appearance of* extra-, intra-,* or combined* extra*hepatic*/intra*hepatic HCCR (*data not shown*). HCCR as a time-dependant covariate was identified by Cox regression analysis as the single strongest hazard for survival (*p* < 0.001; exp⁡(*B*) = 10.156), with no differences between* extra-*,* intra-*, or combined* extra*hepatic*/intra*hepatic locations (Figure 3(b)). Cumulative survival at 5, 10, and 30 years after LT was 80%, 67%, and 45% in HCC recurrence-free patients compared to 28%, 15%, and 10% irrespective of* extra-*,* intra-*, or combined* extra*hepatic*/intra*hepatic locations (Figure 3(b)). Univariate Cox regression analysis of hazards for HCCR (Table 5) revealed a significantly higher risk for HCCR if transplanted outside* hMILAN* (*p* < 0.001, exp⁡(*B*) = 3.645) and a significantly higher risk for HCCR depending on* UICC-7 staging* (*p* < 0.001, Log Rank),* vascular infiltration* (*p* < 0.001, Log Rank), and tumor* grading* (*p* < 0.001, Log Rank).* Underlying diseases* had a significant impact neither on HCC recurrence (*p* > 0.05) (Table 5) and on HCC recurrence related deaths (*p* > 0.05) (Table 6) nor on hospital mortality (*p* > 0.05) and overall mortality (*p* > 0.05) (*data not shown*).* Neoadjuvant therapy* in general did not avoid HCC recurrence (*p* > 0.05) (Table 5) but proved to be significantly advantageous if the tumor had been turned into a complete necrosis (e.g., through PEI, TACE, or RF) or if the tumor had been resected prior to LT (Figures 8 and 9).* Neoadjuvant therapy* did improve survival significantly, if non-HCCR related deaths were excluded from the survival analysis (*p* = 0.024, exp⁡(*B*) = 0.562) (Table 6 and Figure 9(e)). Figures 5 and 6 show that different monomodal/multimodal neoadjuvant treatments had different advantages in relation to the tumor anatomy of the HCC to be treated. Lowest HCC recurrence rates were observed in the group of nondetectable HCCs, which was significantly lower at any time as compared to any other group. Uninodular tumors and unilateral/multinodular tumors had the same cumulative rate of HCC recurrence up to five years after transplantation. Only the follow-up of more than five years revealed further and significant increase of HCC recurrences in unilateral/multinodular tumors as compared to the uninodular group. The highest rate of HCC recurrences was observed in multinodular/bilateral group, which was also significantly higher as compared to the group of multinodular/unilateral HCC. Multinodular unilateral tumors benefited more from PEI whereas multinodular bilateral tumors more likely benefited from TACE. This correlation was found for HCC recurrences as well as for HCC recurrence related deaths (Figures 6(c), 6(d), 7(c), and 7(d)).

Survival was also significantly related to the* UICC-7* staging (Table 6 and Figure 9(b)), meaning that survival decreased with each step-up in UICC-7 staging—with the exception of UICC I and II staged tumors—which had a comparable survival to the reference category of “no or necrotic tumors” (*p* = 0.688, exp⁡(*B*) = 0.746; *p* = 0.402, exp⁡(*B*) = 1.738, resp.). If patients were transplanted outside the* histologic MILAN* criteria, then the HCC recurrence rate was significantly higher (*p* < 0.001, exp⁡(*B*) = 3.507) (Table 5 and Figure 8(c)) and survival significantly deteriorated (*p* < 0.001, exp⁡(*B*) = 4.701) (Table 6 and Figure 9(c)). Vice versa, if transplanted inside* hMILAN* the cumulative survival rate was 72% at 14 years (*p* < 0.001, Log Rank) (Figure 9(c)). Small (V1) and large (V2)* vascular infiltrations* were significant hazards for HCC recurrence (*p* < 0.001, exp⁡(*B*) = 9.050; *p* < 0.001, exp⁡(*B*) = 14.848; resp.) (Table 5 and Figure 8(d)) and HCC recurrence related risks for survival (*p* = 0.001, exp⁡(*B*) = 9.578; *p* < 0.001, exp⁡(*B*) = 14.066; resp.) (Table 6 and Figure 9(d)).

**Figure 10:**
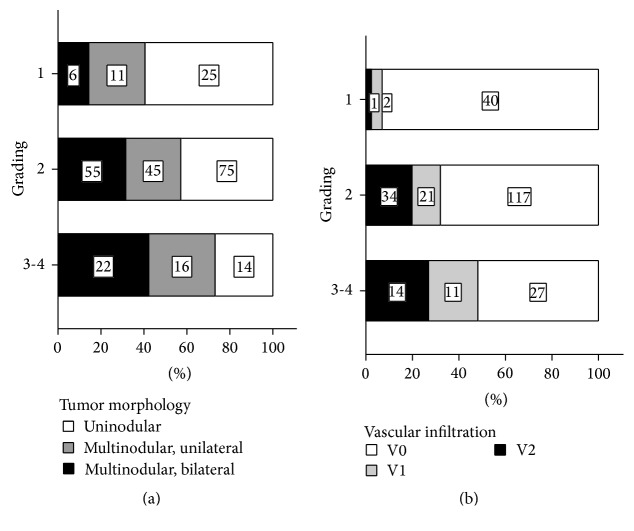
(a) Proportion of* vascular infiltration* with respect to tumor* grading* (G). The incidence of* vascular infiltration* and the proportion of small (V1) and large (V2) infiltrated vessels increased with each step of tumor dedifferentiation (G1 → G2 → G3-4) (*χ* ^2^ *p* = 0.006). The overall incidence of* vascular infiltration* was 7%, 31%, and 48% for G1, G2, and G3-4 graded tumors, respectively. The proportion of small vascular infiltration (V1) was 5%, 12%, and 21% for G1, G2, and G3-4 graded tumors, respectively. The proportion of large vessel infiltration (V2) was 2%, 20%, and 27% for G1, G2, and G3-4 graded tumors, respectively. (b) Proportions of uninodular/multinodular tumors with unilateral/bilateral hepatic spreading in G1–4 graded tumors. There was a close correlation between tumor grading and intrahepatic tumor spreading. The proportion of multinodular and bilateral spread tumors increased with each step of tumor dedifferentiation (G1 → G2 → G3-4) (*χ* ^2^ *p* = 0.016). The proportion of uninodular tumors was 59%, 43%, and 27% for G1, G2, and G3-4 graded tumors, respectively. The proportion of multinodular/unilateral tumors was 26%, 26%, and 31% for G1, G2, and G3-4 graded tumors, respectively. The proportion of multinodular/bilateral tumors was 14%, 31%, and 42% for G1, G2, and G3-4 graded tumors, respectively.

HCCR and survival were both significantly influenced by tumor* grading* (*p* < 0.001,* Log Rank*). The risk for HCCR increased (G2: *p* = 0.018, exp⁡(*B*) = 4.1; G3-4; *p* < 0.001, exp⁡(*B*) = 8.668) (Table 5 and Figure 8(f)) and survival decreased significantly with each step of tumor dedifferentiation (G3-4: *p* = 0.001, exp⁡(*B*) = 10.498) (Table 6 and Figure 9(f)). Furthermore, we found a significant increase in numbers of vascular infiltrating tumors and an increase of large vessel infiltrations per step of tumor dedifferentiation (G1 → G2 → G3-4) (*χ* ^2^ *p* = 0.006) (Figure 10).

Because long-term survival was mainly limited by HCCR (*p* < 0.001, exp⁡(*B*) = 10.156; time-dependent Cox regression) and HCCRs were diagnosed as late as 10 years after LT, but not later than 10.4 years after LT, we aimed to determine the cohort identifiers with respect to this 10.4-year cut-off.

Therefore we analysed the database and compared the group of patients with HCCR occurrence (below 10.4 years) with the group of patients who had HCCR-free follow-up of more than 10.4 years after LT (hospital deaths censored). We found that* hMILAN*,* UICC-7*,* vascular infiltration*, and tumor* grading* were highly significant prognostic parameters (*χ* ^2^ *p* < 0.001, Table 7), while* neoadjuvant therapy* and* underlying diseases* remained nonsignificant.

## 4. Discussion

The results of this study containing the complete data of our center since 1975 demonstrate that hepatocellular carcinoma can be cured by LT—even in advanced tumor stages. As expected, long-term survival was mainly limited by HCC recurrence (HCCR) (*p* < 0.001, exp⁡(*B*) = 10.156; time-dependent Cox regression) and any covariate with high potency for HCC recurrence therefore was a significant negative predictor of survival as well. Vice versa, covariates that were not associated with a significantly higher rate of HCC recurrences (e.g.,* underlying diseases*) had no significant impact on tumor-free survival. We were surprised though to find that not only intrahepatic HCCRs (some of which might have been de novo HCCs) but extrahepatic HCCR also can occur more than 10 years after LT—without synchronous intrahepatic HCC recurrences. We believe that these tumors must have been dormant metastatic HCC manifestations, which existed probably at the time of LT. Thus, it seems that persistent HCC metastasis can reside in extrahepatic locations without being diagnosed or being clinically relevant for many years despite a constant immunosuppressive therapy after transplantation. HCC recurrence-free survival beyond the observed cut-off of 10.4 years' follow–up is a very good prognostic sign independent of the initial tumor staging (e.g., hMILAN and UICC-7 staging) (Table 7). Few patients even were cured from HCCR with observed long-term survival; for example, one patient did survive more than 30 years after repeated resection of lung metastases at one and two years after LT and finally died by natural cause. These findings are only obtainable by long-term observational studies covering at least two decades of follow-up after LT. The fact that even patients with advanced HCCs and tumor stages beyond today's listing criteria did survive for astonishingly long periods of time (as shown by this series of patients) demonstrates the outstanding role of LT in the treatment of HCC.

It is clear that the* histologic MILAN* has no* pre*transplant predictive value, because it is a histological* post*transplantation parameter of the recipient's liver. In this context it is interesting to realize that there was significant proportion of patients who did survive up to 25 years after LT, despite the fact that their tumors had been falsely categorized inside the* iMILAN* classification.

When putting those information together with the knowledge that sensitivity and accuracy of modern imaging techniques have increased over the decades, then one might conclude that the commonly used iMILAN criteria need a revision based on contemporary data. Such an update of iMILAN criteria should take into account that there is—and probably always will be—an existing variance between preoperative* iMILAN* and postoperative* hMILAN*.

Furthermore, for a more accurate assessment of the long-term prognosis, it could be beneficial not only to classify the tumors according to size and numbers of tumors but to consider also the bilateral distribution of tumors on both liver lobes as a prognostic relevant cofactor (Figure 5, Tables 5 and 6).

Hence it is no surprise that several authors already have cast serious doubt [44, 45] on the concept of relying solely on the commonly used iMILAN status for the listing of patients and suggested the extension of the iMILAN criteria, which has already resulted in the definition of alternative listing criteria (e.g., the University of California San Francisco (UCSF) criteria) [46]. But these alternative allocation algorithms also rely solely on pretransplant imaging technology and lack long-term follow-up data that covers at least two decades after LT.

Neoadjuvant therapy in general was only slightly advantageous with respect to HCC recurrence but nevertheless did prolong survival significantly. Because the effect of different neoadjuvant treatment strategies in different patients by different specialists against different tumors of different numbers, sizes, gradings, and status of vascular infiltration is variant, the extent of induced tumor necrosis is completely variant as well. The bottom line is that lowest HCCR rates and best survival rates had been observed when all tumor mass was completely necrotic or missing (e.g., after resection) (Figures 5, 8(b), 8(d), 8(f), 9(b), 9(d), and 9(f)). In other words, the possibly advantageous effect of a neoadjuvant therapy depends on whether all tumor mass is transferred into a complete necrosis or not.

The data further demonstrate that tumor grading (G) is currently an underrated pretransplant prognostic parameter, which seems to be equally relevant for long-term prognosis after LT as compared to allocation algorithms such as iMILAN, which are susceptible for the underrating of relevant histological tumor parameters—for example, the status of vascular infiltration.

Our data also demonstrates the existing close correlation of tumor dedifferentiation with intrahepatic tumor spreading (Figure 10(a)) and the potency of tumor cell differentiation (*grading*, G) to predict vascular infiltration (Figure 10(b)). As tumor grading and vascular infiltration have a significant prognostic impact on HCC recurrence and patient survival, these cofactors should be routinely utilized for a better timing of LT in HCC patients.

## 5. Conclusion

Our retrospective data analysis demonstrates the historical evolution in liver transplantation from the 1970s until today. We clearly show that the diagnosis of hepatocellular carcinoma can be survived for the long-term after liver transplantation (LT).* Vascular infiltration* is one decisive predictor of HCCR and a major hazard for survival but is not easily and reliably detectable before LT. Furthermore, the data shows that* grading* is closely related to* vascular infiltration* and a* multinodular* and* bilateral tumor spreading*. Grading can be easily and reliably determined prior to LT by biopsy. We believe that this observation should be taken into account in liver allocation and the timing of LT. Biopsies could be well acquired synchronously during RFA or PEI bridging interventions. Furthermore, due to the fact that needle tract seeding has a very low incidence of only 0.13% [47] and in face of the potential benefits we believe that repeated fine needle biopsies [48, 49] of HCC tumors should be considered while the patient is listed for LT. One thinkable scenario though might be that a detected dedifferentiation would trigger a drop-out from the waiting list due to expected poor prognosis and the implied ethical and judicial dilemma for patients who may remove themselves from the liver transplant waiting list by agreeing to the consequences of liver biopsy cannot be easily resolved. Vice versa, a consequence of more positive thinking could be a faster donor liver allocation process in case of detected progressive cellular dedifferentiation, hoping to perform LT before vascular infiltration and metastatic seeding of HCC have taken place. Of course, a single biopsy provides no complete picture of the entire tumor, especially not if the tumor has a multinodular morphology with different tumor gradings in each tumor nodule. However, our data show that every single detected dedifferentiation represents a significant risk increment for HCC recurrence and therefore should be considered accordingly, not only during the initial listing of patients, but also in patients who are already listed and waiting for a donor organ.

Overall, we believe that an updated and refined liver allocation score for HCC patients could be developed to gain a higher predictive power compared to the usual* iMILAN* classification. Further refined biometrical studies on this issue are in progress.

## Figures and Tables

**Table 1:** In- and excluded subpopulations for the analysis of HCC recurrence and HCC recurrence related deaths.

Subpopulations (hospital mortality excluded)	Cox regression analysis for the risk of		HCC recurrence	
	HCC recurrence (Table 5)	HCC recurrence related deaths (Table 6)	No	Yes
Alive, *n* = 136	Included	Included	125	11
Deaths caused by HCCR, *n* = 63	Included	Included	0	63
Deaths *not* caused by HCCR, *n* = 52	Included	**Excluded**	43	9
Overall	251	199	168	83

**Table 2:** Descriptive statistics.

Underlying disease	Gender		Age at LT [year]				Tumor morphology				Tumor volume [cm^3^]				AFP [ng/mL]				Graft type					CIT [min.]				LT #			
	f	m	Mean	Median	Max.	Min.	No tumor detectable	Uninodular	Multinodular, unilateral	Multinodular, bilateral	Mean	Median	Max.	Min.	Mean	Median	Max.	Min.	Full size	Partial		Split		Mean	Median	Max.	Min.	1st	2nd	3rd	Total
																				Deceased	Living	Deceased	Living								
Hepatitis B with D	1	14	50,6	50,3	61,6	38,6	1	9	2	3	86	8	978	—	418	18,3	5571	1	13	—	2	—	—	531	507	880	263	14	1	—	15
Hepatitis B	7	78	50,7	53,8	69,7	21,6	5	32	23	25	236	42	4849	—	2405	29	176659	1	78	1	2	4	—	638	635	1577	207	78	7	—	85
Hepatitis C with B	2	10	51,2	57,1	65,2	8,1	1	7	3	1	30	13	202	—	3478	25,5	32000	5	12	—	—	—	—	607	518	1054	388	11	1	—	12
Hepatitis C	20	66	54,2	54,7	66,7	25	5	40	25	16	80	16	1164	—	839	21	56100	1	74	—	4	6	2	584	571	1302	100	77	8	1	86
Hepatoblastoma	3	1	14,8	14,1	17,8	13,3	—	1	—	3	432	375	905	74	304	304	600	7	4	—	—	—	—	866	724	1740	275	4	—	—	4
Adenomatosis	3	1	41,7	40,7	56,3	29,2	—	1	2	1	27	6	93	4	4,75	5	7	2	4	—	—	—	—	638	579	1122	271	3	1	—	4
Hypertyrosinemia	—	2	14,4	14,4	19,4	9,3	—	2	—	—	34	34	34	34	1276	1276	2431	120	2	—	—	—	—	420	420	480	359	2	—	—	2
Wilson's disease	1	—	43,8	—	—	—	—	1	—	—	34	—	—	—	5	—	—	—	1	—	—	—	—	572	572	572	572	1	—	—	1
Hemochromatosis	—	4	56,8	56,7	63,5	50,3	—	—	2	2	452	19	1768	2	248	247	485	13	4	—	—	—	—	635	618	786	516	3	1	—	4
*α*1 antitrypsin def.	1	—	56,8	—	—	—	—	1	—	—	17	—	—	—	1	—	—	—	1	—	—	—	—	588	588	588	588	1	—	—	1
Budd Chiari	1	1	36,2	36,2	40,3	32,2	—	1	1	—	263	263	525	2	39	39	73	5	2	—	—	—	—	665	665	773	557	1	1	—	2
Alcohol abuse	6	41	55,4	55,4	68,7	26	4	15	10	18	534	34	14137	—	3679	12,3	109718	1	45	—	1	1	—	674	629	1970	187	42	5	—	47
Androgen therapy	—	1	29,6	—	—	—	—	—	—	1	13	—	—	—	5	—	—	—	1	—	—	—	—	552	552	552	552	1	—	—	1
Biliary cirrhosis	3	—	24,9	17,7	54,1	3	—	2	—	1	131	9	382	3	39	39,3	39,3	39	1	—		2	—	644	773	827	332	3	—	—	3
Cryptogenic cirrhosis	23	27	49	51,8	67	18,6	4	21	9	16	856	93	8181	—	6100	10,5	214975	1	44	1	1	4	—	628	636	1180	227	46	4	—	50
Autoimmune hepatitis	—	1	72	—	—	—	—	—	1	—	38	—	—	—	485	—	—	—	1	—	—	—	—	971	971	971	971	1	—	—	1
Chronic lead intox.	—	1	44,7	—	—	—	—	—	1	—	1216	—	—	—	540	—	—	—	—	1	—	—	—	865	865	865	865	1	—	—	1

**Table 3:** Rate and anatomical sites of HCC recurrences with respect to tumor morphology and neoadjuvant therapy.

		HCC						
		Not detectable	Uninodular		Multinodular, unilateral		Multinodular, bilateral	
		Neoadjuvant therapy	Neoadjuvant therapy		Neoadjuvant therapy		Neoadjuvant therapy	
			No	Yes	No	Yes	No	Yes
Counts/%	*n*	*n* %	*n* %	*n* %	*n* %	*n* %	*n* %	*n* %
HCC recurrence								
No	230	**19 **95	**53 **83	**55 **80	**25 **68	**32 **76	**22 **49	**24 **57
Yes	89	**1 **5	**11 **17	**14 **20	** 12 **32	**10 **24	**23 **51	**18 **43
Abdominal wall	2			**2**				
Adrenal	1						**1**	
Bone	5			**2**			**2**	**1**
Bone + abdominal wall	2		**1**		**1**			
Bone + liver	1							**1**
Brain	2	**1**	**1**					
Esophagus	1		**1**					
Liver	17		**2**	**2**	**5**	**2**	**4**	**2**
Liver + abdominal wall	2						**1**	**1**
Liver + adrenal	2						**1**	**1**
Liver + diaphragm	1						**1**	
Liver + lung	5			**1**			**2**	**2**
Liver + lung + bone	1							**1**
Liver + lung + kidney	1			**1**				
Liver + lymph node	3			**1**	**1**			**1**
Liver + peritoneum	1						**1**	
Lung	20		**2**		**2**	**4**	**7**	**5**
Lung + bone	4			**3**			**1**	
Lung + bone + brain	1						**1**	
Lung + brain	4				**2**			**2**
Lung + liver + lymph node + abdominal wall	1					**1**		
Lymph node	6		**1**		**1**	**2**	**1**	**1**
Peritoneum	5		**3**	**2**				
Peritoneum + bone	1					**1**		

**Table 4:** Tumor morphology, neoadjuvant therapy, tumor response to neoadjuvant therapy, HCC recurrence rate, and HCC recurrence related deaths.

Tumor morphology	Neoadjuvant therapy	Response			HCC recurrence		HCC recurrence related death		
		Vital tumor remnants	Full-necrotic	No tumor detectable	No	Yes	No	Yes	Not specified
No tumor	PEI	—	—	2 100%	2 100%	—	2 100%	—	—
	TACE	—	—	2 100%	2 100%	—	2 100%	—	—
	Surgery	—	—	9 100%	8 89%	1 11%	8 89%	1 11%	—
	Surgery + TACE	—	—	2 100%	2 100%	—	2 100%	—	—
	Surgery + TACE + CTX	—	—	1 100%	1 100%	—	1 100%	—	—
	Overall	*0 *	*0 *	*16 *	*15 *	*1 *	*15 *	*1 *	*0 *

Uninodular	None	48 100%	—	—	37 77%	11 23%	39 81%	8 17%	1 2%
	PEI	22 73%	8 27%	—	25 83%	5 17%	25 83%	4 13%	1 3%
	TACE	13 81%	3 19%	—	12 75%	4 25%	13 81%	3 19%	—
	PEI + TACE	6 75%	2 25%	—	7 88%	1 13%	8 100%	—	—
	Surgery	3 75%	1 25%	—	—	4 100%	2 50%	2 50%	—
	CTX + TACE	2 67%	1 33%	—	3 100%	—	3 100%	—	—
	RFA	2 100%	—	—	2 100%	—	2 100%	—	—
	RFA + TACE	—	1 100%	—	1 100%	—	1 100%	—	—
	PEI + RFA	—	1 100%	—	1 100%	—	1 100%	—	—
	Overall	*96 *	*17 *	*0 *	*88 *	*25 *	*94 *	*17 *	*2 *

Multinodular, unilateral	None	25 100%	—	—	14 56%	11 44%	17 68%	8 32%	—
	PEI	5 83%	1 17%	—	6 100%	—	6 100%	—	—
	TACE	6 86%	1 14%	—	4 57%	3 43%	5 71%	2 29%	—
	PEI + TACE	4 100%	—	—	4 100%	—	4 100%	—	—
	Surgery	3 100%	—	—	1 33%	2 67%	2 67%	1 33%	—
	CTX	4 100%	—	—	2 50%	2 50%	4 100%	—	—
	Surgery + TACE	3 100%	—	—	1 33%	2 67%	1 33%	2 67%	—
	RFA	3 100%	—	—	2 67%	1 33%	2 67%	1 33%	—
	Surgery + TACE + CTX	1 100%	—	—	1 100%	—	1 100%	—	—
	RFA + TACE	1 100%	—	—	1 100%	—	1 100%	—	—
	Overall	*55 *	*2 *	*0 *	*36 *	*21 *	*43 *	*14 *	*0 *

Multinodular, bilateral	None	34 100%	—	—	12 35%	22 65%	15 44%	19 56%	—
	PEI	4 100%	—	—	4 100%	—	4 100%	—	—
	TACE	7 100%	—	—	2 29%	5 71%	2 29%	5 71%	—
	PEI + TACE	6 75%	2 25%	—	5 63%	3 38%	6 75%	2 25%	—
	Surgery	2 100%	—	—	—	2 100%	—	2 100%	—
	CTX	2 100%	—	—	—	2 100%	1 50%	1 50%	—
	Surgery + TACE	2 100%	—	—	1 50%	1 50%	1 50%	1 50%	—
	Surgery + TACE + CTX	3 100%	—	—	3 100%	—	3 100%	—	—
	Surgery + CTX	1 100%	—	—	—	1 100%	—	1 100%	—
	PEI + RFA	1 100%	—	—	—	1 100%	1 100%	—	—
	Surgery + PEI	1 100%	—	—	—	1 100%	1 100%	—	—
	Overall	*63 *	*2 *	*0 *	*27 *	*38 *	*34 *	*31 *	*0 *

Total		**214**	**21**	**16**	**166**	**85**	**186**	**63**	**2**

**Table 5:** Identification of hazards for HCC recurrence by univariate Cox regression, *n* = 251 (hospital deaths excluded).

Univariate Cox regressions for HCC recurrence		*n*	*p*	exp(*B*)/hazard	95.0% CI	
					Lower	Upper
Underlying disease	*Hepatitis B with D*	12	0.176	*Reference category*		
	Hepatitis B	64	0.405	1.667	0.5	5.555
	Hepatitis C	75	0.847	1.126	0.336	3.777
	Hepatitis C with B	9	0.419	0.394	0.041	3.784
	Alcohol	33	0.131	2.603	0.752	9.003
	Cryptogenic cirrhosis	38	0.375	1.76	0.505	6.128
	Other	20	0.417	1.732	0.459	6.53

Tumor vitality	*Vital tumor*	*214*	0.048	*Reference category*		
	Full-necrotic tumor	21	0.118	0.399	0.126	1.263
	No tumor detectable	16	0.053	0.142	0.02	1.022

Tumor morphology	*No tumor detectable*	16	<0.001	*Reference category*		
	Uninodular	113	0.192	3.789	0.513	27.971
	Multinodular unilateral	57	0.073	6.251	0.840	46.513
	Multinodular bilateral	65	0.008	14.505	1.990	105.733

UICC-7	*No or necrotic tumor*	37	<0.001	*Reference category*		
	UICC I	69	0.646	0.75	0.219	2.563
	UICC II	71	0.200	2.041	0.686	6.073
	UICC IIIA	25	<0.001	7.428	2.513	21.959
	UICC IIIB	32	<0.001	13.734	4.759	39.631
	UICC IIIC	7	0.001	9.808	2.627	36.611
	UICC IVA	7	<0.001	54.098	14.542	201.253
	UICC IVB	3	<0.001	180.683	34.823	937.506

hMILAN	*Inside*	*148*	*Reference category*			
	Outside	103	<0.001	3.507	2.237	5.496

Vascular infiltration	*No or necrotic tumor*	37	<0.001	*Reference category*		
	V0	148	0.254	1.829	0.648	5.161
	V1	28	<0.001	9.05	3.042	26.92
	V2	37	<0.001	14.848	5.206	42.353
	Missing data	1	0.977	0	0	1.65*E* + 245

Neoadj. therapy	No	107	*Reference category*			
	Yes	144	0.071	0.676	0.441	1.035

Grading	*No or necrotic tumor*	37	<0.001	*Reference category*		
	G1	35	0.272	1.937	0.596	6.299
	G2	130	0.023	3.282	1.179	9.138
	G3-4	43	<0.001	6.672	2.313	19.249
	Missing data	6	0.008	6.550	1.636	26.214

HCC = hepatocellular carcinoma.

UICC-7 = 7th edition TNM classification of Unité International Contre Cancer.

hMILAN = histologic MILAN classification.

Vascular infiltration: V0 = none, V1 = small vessels, and V2 = large vessels.

Tumor grading: G1 = low, G2 = intermediate, and G3-4 = high to anaplastic.

**Table 6:** Identification of hazards for HCC recurrence related deaths by univariate Cox regression, *n* = 199 (non-HCC recurrence related deaths excluded).

Univariate Cox regressions for HCC recurrence related deaths		*n*	*p*	exp(*B*)/hazard	95.0% CI	
					Lower	Upper
Underlying disease	Hepatitis B with D	11	0.348	*Reference category*		
	Hepatitis B	50	0.368	1.964	0.451	8.553
	Hepatitis C	55	0.542	1.584	0.361	6.939
	Hepatitis C with B	6	0.908	0.868	0.079	9.588
	Alcohol	30	0.162	2.919	0.65	13.109
	Cryptogenic cirrhosis	30	0.13	3.151	0.714	13.896
	Other	17	0.433	1.93	0.373	9.979

Tumor vitality	Vital tumor	168	0.083	*Reference category*		
	Full-necrotic tumor	17	0.147	0.352	0.086	1.442
	No tumor detectable	14	0.084	0.175	0.024	1.264

Tumor morphology	*No tumor detectable*	14	<0.001	*Reference category*		
	Uninodular	89	0.323	2.765	0.368	20.791
	Multinodular unilateral	41	0.104	5.354	0.707	40.567
	Multinodular bilateral	55	0.019	10.898	1.488	79.801

UICC-7	*No or necrotic tumor*	31	<0.001	*Reference category*		
	UICC I	54	0.688	0.746	0.178	3.124
	UICC II	51	0.402	1.738	0.477	6.327
	UICC IIIA	21	0.003	6.771	1.944	23.584
	UICC IIIB	26	<0.001	12.792	3.791	43.16
	UICC IIIC	6	0.006	8.066	1.800	36.142
	UICC IVA	7	<0.001	226.972	46.041	1118.915
	UICC IVB	3	<0.001	91.043	16.824	492.692

hMILAN	Inside	112	*Reference category*			
	Outside	87	<0.001	4.701	2.700	8.185

Vascular infiltration	*No or necrotic tumor*	31	<0.001	*Reference category*		
	V0	117	0.371	1.733	0.52	5.779
	V1	21	<0.001	9.578	2.769	33.128
	V2	30	<0.001	14.066	4.221	46.866

Neoadj. therapy	No	82	*Reference category*			
	Yes	117	0.010	0.525	0.321	0.859

Grading	*No or necrotic tumor*	31	<0.001	*Reference category*		
	G1	26	0.26	2.179	0.562	8.442
	G2	103	0.061	3.098	0.948	10.124
	G3-4	36	0.001	7.909	2.357	26.542
	Missing data	3	0.007	11.921	1.980	71.774

HCC = hepatocellular carcinoma.

UICC-7 = 7th edition TNM classification of Unité International Contre Cancer.

hMILAN = histologic MILAN classification.

Vascular infiltration: V0 = none, V1 = small vessels, and V2 = large vessels.

Tumor grading: G1 = low, G2 = intermediate, and G3-4 = high to anaplastic.

**Table 7:** Distribution of covariates and subcategories in HCCR versus HCCR-free postoperative episodes (cut-off time 10.4 years) (*n* = 115) (hospital deaths and patients without HCCR within 10.4 years excluded).

Main category	Overall (*n* = 115) (%)	Subcategory	Follow-up		*χ* ^2^
			<10.4 years (*n* = 82)	>10.4 years (*n* = 33)	*p* value
Underlying disease	7 (6.1%)	Hepatitis B with D	3 (3.7%)	4 (12.1%)	*0.076*
	35 (30.4%)	Hepatitis B	23 (28.0%)	12 (36.4%)	
	30 (26.1%)	Hepatitis C	20 (24.4%)	10 (30.3%)	
	3 (2.6%)	Hepatitis C with B	1 (1.2%)	2 (6.1%)	
	15 (13.0%)	Alcohol	14 (17.1%)	1 (3.0%)	
	16 (13.9%)	Cryptogenic	13 (15.9%)	3 (9.1%)	
	9 (7.8%)	Other	8 (9.8%)	1 (3.0%)	

UICC-7	9 (7.8%)	No or necrotic tumor	3 (3.7%)	6 (18.2%)	*0.000*
	19 (16.5%)	I	7 (8.5%)	12 (36.4%)	
	27 (23.5%)	II	16 (19.5%)	11 (33.3%)	
	18 (15.7%)	IIIA	18 (22.0%)	0	
	27 (23.5%)	IIIB	25 (30.5%)	2 (6.1%)	
	7 (6.1%)	IIIC	5 (6.1%)	2 (6.1%)	
	5 (4.3%)	IVA	5 (6.1%)	0	
	3 (2.6%)	IVB	3 (3.7%)	0	

hMILAN	50 (43.5%)	Inside	27 (32.9%)	23 (69.7%)	*0.000*
	65 (56.5%)	Outside	55 (67.1%)	10 (30.3%)	

Before treatment	64 (55.7%)	No	43 (52.4%)	21 (63.6%)	*0.274*
	51 (44.3%)	Yes	39 (47.6%)	12 (36.4%)	

Vascular infiltration	9 (7.8%)	No or necrotic tumor	3 (3.7%)	6 (18.2%)	*0.000*
	55 (47.8%)	0	32 (39.0%)	23 (69.7%)	
	19 (16.5%)	1	17 (20.7%)	2 (6.1%)	
	32 (27.8%)	2	30 (36.6%)	2 (6.1%)	

Grading (G)	9 (8.3%)	No or necrotic tumor	3 (2.8%)	6 (5.5%)	*0.000*
	20 (18.3%)	1	9 (8.3%)	11 (10.1%)	
	52 (47.7%)	2	42 (38.5%)	10 (9.2%)	
	28 (25.7%)	3-4	24 (22.0%)	4 (3.7%)	

HCC = hepatocellular carcinoma.

UICC-7 = 7th edition TNM classification of Unité International Contre Cancer.

hMILAN = histologic MILAN classification.

Vascular infiltration: V0 = none, V1 = small vessels, and V2 = large vessels.

Tumor grading: G1 = low, G2 = intermediate, and G3-4 = high to anaplastic.

## Abbreviations

LT: Liver transplantation
HCC: Hepatocellular carcinoma
HCCR: Hepatocellular carcinoma recurrence
hMILAN: Histology-based MILAN
iMILAN: Imaging-based MILAN.
